# HIF-1 Has a Central Role in *Caenorhabditis elegans* Organismal Response to Selenium

**DOI:** 10.3389/fgene.2020.00063

**Published:** 2020-02-25

**Authors:** Laura Romanelli-Credrez, Maria Doitsidou, Mark J. Alkema, Gustavo Salinas

**Affiliations:** ^1^ Laboratorio de Biología de Gusanos. Unidad Mixta, Departamento de Biociencias, Facultad de Química, Universidad de la República–Institut Pasteur de Montevideo, Montevideo, Uruguay; ^2^ Centre for Discovery Brain Sciences (CDBS), University of Edinburgh, Edinburgh, United Kingdom; ^3^ Neurobiology Department, University of Massachusetts Medical School, Worcester, MA, United States

**Keywords:** selenium, selenite, stress, HIF-1, EGL-9, CYSL-1, sulfide, *Caenorhabditis elegans*

## Abstract

Selenium is a trace element for most organisms; its deficiency and excess are detrimental. Selenium beneficial effects are mainly due to the role of the 21^st^ genetically encoded amino acid selenocysteine (Sec). Selenium also exerts Sec-independent beneficial effects. Its harmful effects are thought to be mainly due to non-specific incorporation in protein synthesis. Yet the selenium response in animals is poorly understood. In *Caenorhabditis elegans*, Sec is genetically incorporated into a single selenoprotein. Similar to mammals, a 20-fold excess of the optimal selenium requirement is harmful. Sodium selenite (Na_2_SeO_3_) excess causes development retardation, impaired growth, and neurodegeneration of motor neurons. To study the organismal response to selenium we performed a genetic screen for *C. elegans* mutants that are resistant to selenite. We isolated non-sense and missense *egl-9/EGLN* mutants that confer robust resistance to selenium. In contrast, *hif-1*/*HIF* null mutant was highly sensitive to selenium, establishing a role for this transcription factor in the selenium response. We showed that EGL-9 regulates HIF-1 activity through VHL-1, and identified CYSL-1 as a key sensor that transduces the selenium signal. Finally, we showed that the key enzymes involved in sulfide and sulfite stress (sulfide quinone oxidoreductase and sulfite oxidase) are not required for selenium resistance. In contrast, knockout strains in the persulfide dioxygenase ETHE-1 and the sulfurtransferase MPST-7 affect the organismal response to selenium. In sum, our results identified a transcriptional pathway as well as enzymes possibly involved in the organismal selenium response.

## Introduction

Selenium (Se) is an essential trace element in animals. Se deficiency and excess are detrimental to organismal fitness. In most species, including mammals, the adequate range between deficient, essential, and toxic Se supply is particularly narrow (Combs, 2001). In mammals, Se is important for proper function of the thyroid, male reproduction-, cardiovascular-, and immune-system functions (Schoenmakers et al., 2010; Labunskyy et al., 2014; Loscalzo, 2014; Schoenmakers et al., 2016) due to selenocysteine (Sec)-containing proteins (Kryukov et al., 2003; Labunskyy et al., 2014). At the organismal level, Se toxicity is observed at 20 times the dietary requirement (Wilber, 1980; O'Dell and Sunde, 1997). The adverse effects of Se excess have been associated with altered thiol metabolism, redox imbalance, oxidative stress, and protein folding (Wilber, 1980; O'Dell and Sunde, 1997). It is thought that Se deleterious effects are due to Se-derived metabolites and misincorporation of Sec and selenomethionine (SeMet) during protein synthesis at cysteine and methionine sites, respectively (Mézes and Balogh, 2009; Hoffman et al., 2019).

Sec biosynthesis, coding, and decoding are well understood, as well as the function of several selenoprotein families (Leinfelder et al., 1988; Böck et al., 1991; Berry, 2005; Labunskyy et al., 2014). Yet, the mechanisms and pathways associated with Se metabolism and toxicity are poorly understood. While supernutritional levels of Se can be toxic, supplementation with selenite has been implemented in Se-deficient areas and also used as cancer therapeutics (Combs, 2001). Understanding the genetic basis of adaptation to levels of Se can provide insights into the nutritional and toxicological aspects of this trace element.

*Caenorhabditis elegans* is a genetically tractable experimental model suited to understand Se biology *in vivo*. Similar to mammals, Se is a trace element for *C. elegans*. Genes required for Sec biosynthesis and incorporation into proteins are conserved, and dedicated to a single selenoprotein, the cytosolic thioredoxin reductase, TRXR-1 (Taskov et al., 2005). Previous studies reported that trace amounts of selenite exert multiple beneficial effects on development, fertility, cholinergic signaling (Li et al., 2011) and oxidative stress resistance in *C. elegans* (Li et al., 2014b). A proposed mechanism for the role of selenite in oxidative stress resistance involves the activation of the transcription factor DAF-16/*FOXO*. It was demonstrated that low amounts of selenite result in a DAF-16/*FOXO* nuclear translocation and increased expression of DAF-16 target genes, such as the superoxide dismutase encoding gene *sod-3* (Li et al., 2014b). Recent work reported that selenite enhances the innate immune response against *C. elegans* pathogen *Pseudomonas aeruginosa* PA14 *via* SKN-1/*NRF2* transcription factor (Li et al., 2014a). On the other hand, high concentrations of Se are detrimental to *C. elegans*. Several studies have shown that exposure to sodium selenite induces oxidative stress, causes development retardation, impaired growth, and neurodegeneration of cholinergic and GABAergic motor neurons and finally muscular alterations (Morgan et al., 2010; Estevez et al., 2012; Estevez et al., 2014). These effects lead to progressive motility loss, culminating in irreversible paralysis. In both *C. elegans* and mammals, neurons are particularly susceptible to Se imbalance (Vinceti et al., 2001; Schweizer, 2016), reinforcing the utility of this model.

Most Se toxicity studies have been performed with sodium selenite (Morgan et al., 2010; Boehler et al., 2013; Li et al., 2014c), and its biotransformations are not completely understood. A recent study found that selenite was the only chemical species found in worms exposed to this compound (Boehler et al., 2013; Rohn et al., 2018). Selenite reduction has been proposed to be performed by thioredoxin reductase (TRXR) (Bjornstedt and Kumar, 1992; Turner et al., 1998). However, in *C. elegans* neither single TRXR-1 and TRXR-2 mutants nor the double TRXR-1; TRXR-2 mutant differed in Se sensitivity from the wild type (Boehler et al., 2013; Rohn et al., 2018). Transcriptomic experiments showed that under elevated Se concentrations, the expression of oxidoreductase genes was enriched suggesting an increase in reactive oxygen species (Boehler et al., 2014).

To identify genes required for organismal Se response, we performed a screen for selenite resistance. As a result of chemical mutagenesis and selection of Se-resistant strains, we isolated different mutants in *egl-9*, a HIF-1 prolyl hydroxylase. EGL-9/*EGLN* negatively regulates the transcriptor factor HIF-1/*HIF*, a master regulator of the hypoxia response in different organisms (Wang and Semenza, 1995; Epstein et al., 2001; Semenza, 2004). In *C. elegans*, this transcription factor is central to the organismal response to hypoxia, hydrogen sulfide (H_2_S), and iron levels, as well as to several metabolic cues and stressors (Semenza, 2004; Budde and Roth, 2010; Wong et al., 2013). Our results indicated that HIF-1 is a key transcription factor in the Se organismal response and provided evidence regarding Se sensor and effectors involved in this pathway.

## Materials and Methods

### *C. elegans* Strains and Culture Conditions

The general methods used for culturing and maintenance of *C. elegans* are described in (Brenner, 1974). The wild-type strain used in this study was *C. elegans* Bristol N2 (N2). Strains were obtained from the *Caenorhabditis* Genetic Center and the *C. elegans* National Bioresource Project of Japan. **Supplementary Table 1** describes all the strains used in this study detailing the genotype and the source.

### Non-Clonal F2 Mutant Screen for Sodium Selenite Resistant Mutants

N-ethyl-N-nitrosourea (ENU) mutagenesis was performed as described in (Jorgensen and Mango, 2002) with some modifications. N2 animals from six plates (9 cm) were incubated with ENU for 4 h. Animals were washed and placed in OP50-seeded NGM plates. About 150 L4 worms were transferred to two plates, and allowed to grow over night (P0). P0 animals were allowed to lay eggs for 9 h, transferring worms to fresh plates after 3 h. F1 animals were allowed to grow and lay eggs. When the first F2 larvae hatched, the F1 were washed off the plates. Around 5,400 haploid genomes were screened.

For the screen for sodium selenite resistant animals, two protocols were used: 1: F2 worms were allowed to grow to young adults and transferred to plates with 10 mM of sodium selenite. After 72 h, healthy animals were recovered to a fresh NGM plate, singled and re-tested for survival three times. As a result, five mutants were isolated and the strongest penetrant strain (more adult animals alive after 72 h in sodium selenite 10 mM) was further characterized (QW1264). 2: Half of the F2 adult worms were bleached to generate synchronized F3 animals. The F3 embryos were exposed to 5 mM of sodium selenite for 96 h. Animals in the L3 stage were singled and re-tested for survival three times. Nine mutants were isolated from this procedure. The strongest penetrant strain was further characterized (QW1263).

### Determination of Modes of Inheritance

To determine the mode of inheritance (autosomal/X-linked and dominance/recessiveness) of mutation/s in QW1263 and QW1264 mutants, we performed crosses with the wild-type strain. F1 males and hermaphrodites were examined in selenite 10 mM. Additionally, 10 F1 were isolated and the F2 examined in selenite.

### Whole-Genome Sequencing and Data Analysis

For the mutation mapping, we followed the “Variant Discovering Mapping” method as described in (Doitsidou et al., 2016). The mutant is crossed with the original strain used for mutagenesis and a pool of recombinant F2 are selected by the studied phenotype. Once several F2 mutant homozygous recombinant animals were identified, they were analyzed three times for the Se resistance phenotype to confirm the homozygosis. Fifteen and 12 independent recombinant F2 animals were isolated for QW1263 and QW1264, respectively.

Worms were grown until they were gravid adults, then they were harvested, pooled, and washed several times. Animals were left for 2 h with gentle shaking to purge them of bacteria. Finally, worms were washed and 500 μL pelleted worms were stored at −80°C until further use.

For DNA extraction, the protocol of Gentra Puregene Kit (Qiagen) was followed.

Raw data processing was performed using several modules of the Galaxy platform. The pipeline Cloudmap Unmapped Mutant Workflow was used for alignment of the sequencing reads to the reference genome and variant calling. The pipeline Cloudmap Variant Discovery Mapping was used for the SNP mapping analysis (Minevich et al., 2012).

### Generation of Transgenic Animals

Transgenic lines were obtained according to (Mello et al., 1991). The pCFJ90 plasmid containing the injection marker P*myo-2*::*mCherry::unc-54utr* (5 ng/µl) was co-injected with constructs containing P*hif-1*::*hif-1*::*gfp* and P*vhl-1*::*vhl-1*::*gfp* (30 ng/µl) cloned into the pPD95.77 plasmid and injected into ZG31 [*hif-1(ia04)*] and CB5602 [*vhl-1(ok161)*] animals, respectively. From the progeny of the injected animals, three independent transgenic lines that stably transfer extrachromosomal arrays to the progeny were selected.

### Selenium Toxicity Tests

#### Toxicity Tests in Solid Media Plates

Different amounts of sodium selenite were added to NGM media before pouring plates to obtain 2, 5, 10, and 20 mM final concentrations. To avoid possible bacterial metabolic interference, heat-killed OP50 was used as a food source. For this purpose, a 20 X concentrated bacteria culture was incubated at 65°C for 30 min. Fifty μL of killed bacteria was added to the center of NGM plates (5 cm) and allowed to dry. Forty-fifty L4-young adult worms were transferred to plates with selenite, and the number of living and dead worms was quantified. Every day alive animals were transferred to new selenite plates. At least three independent experiments were performed.

#### Toxicity Tests in Liquid Media Using the Infrared Tracking Device WMicrotracker

The toxicity was measured using the infrared tracking device WMicrotrackerTM ONE (PhylumTech, Santa Fe, Argentina). The method used to assess motility is described in detail in (Simonetta and Golombek, 2007). Briefly, the system detects motility through the interference to an array of infrared light microbeams, caused by worm movement.

The readout is counts per unit of time (15 min). Each count represents the interruption of an infrared beam by worms. Experiments were performed in 96 well plates, using 80 synchronized L4 animals per well in a final volume of 100 µL. Four wells per condition per strain were assessed in each replica. Experiments were repeated at least three times.

In all cases the counts per well at different times are normalized by the counts before adding the compound of interest or its vehicle (basal counts). To this basal activity is assigned an arbitrary value of one. The normalization corrects for minor differences due to the number of worms per well. This parameter (counts treated or vehicle/basal counts) is referred to as locomotor activity. All the assays include the wild-type strain and vehicle for each strain as controls.

### RNAi Experiments

The interference of *cysl-3* and *suox-1* expression were performed in the N2 strain by feeding worms with bacteria expressing double strain RNA (dsRNA) of the genes of interest, as described in (Kamath et al., 2000) with RNAi clones JA:R08E5.2 and JA:H13N06.4.

RNAi treated worms (F3 generation) were transferred to NGM plates with sodium selenite (5 mM). The number of living worms was quantified after 24 h. *E. coli* HT115 encoding the dsRNA of *dpy-11* as well as bacteria with the empty vector were used as interference positive and negative controls, respectively. In the case of *suox-1*, interfered animals also were exposed to sodium sulfite (0.5 g/L) as an additional control.

### Statistical Analysis

Normality and variance homogeneity were determined by Shapiro-Wilk and Levene's test, respectively, with a 5% of significance level. Normal data were compared by ANOVA test and subsequent Tukey's test for pairwise comparisons. Samples with unequal variances were compared using Welch F test and Tukey's test for pairwise comparisons. Non-parametric data were compared using Kruskal-Wallis test and Mann-Whitney pairwise post-hoc test.

## Results

### *egl-9* Mutants Are Resistant to Selenite

**Figure 1:**
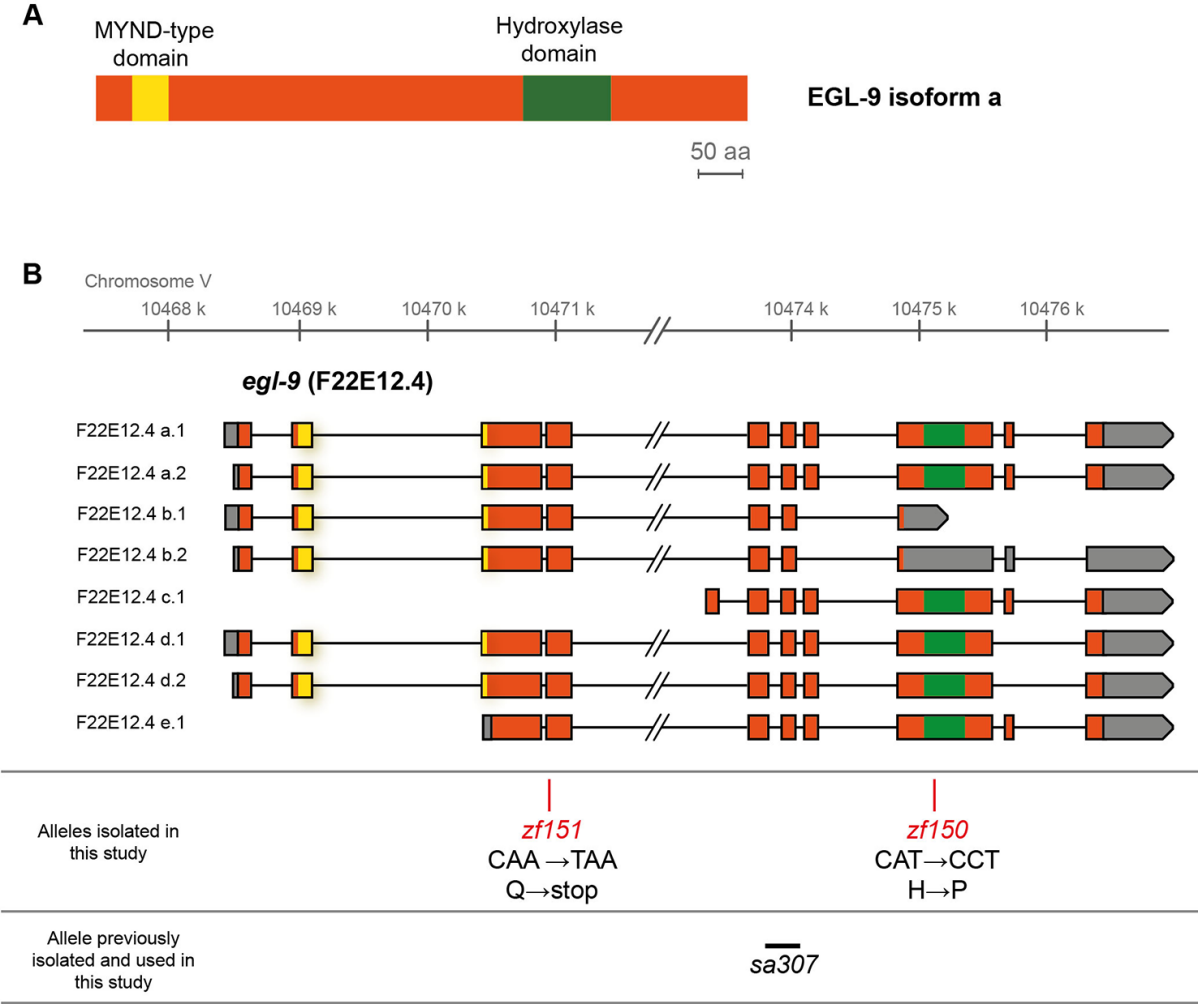
EGL-9 protein domains and *C. elegans egl-9* transcripts representation. **(A)** Scheme of the primary structure of EGL-9 protein (isoform a), highlighting the regions that constitute the hydroxylase and MYND domains. aa: amino acids. **(B)** Different *egl-9* transcripts reported (F22E12.4a-e). The coding region is represented in orange, UTR sequences in gray and introns as lines. The coding regions for the hydroxylase domain and the MYND domain are indicated in green and yellow, respectively. The position and identity of *egl-9* mutant alleles isolated in this study (allele *zf150* and *zf151*), as well as the location of the previously reported *egl-9(sa307)* allele (243 bp deletion) are indicated.

To search for genes involved in Se metabolism, we performed a genetic screen for Se resistant mutants. Approximately 5,500 mutant haploid genomes were screened for sodium selenite resistance. F2 mutagenized adults and F3 mutagenized embryos were exposed to 10 and 5 mM of sodium selenite, respectively. From each screen, the most resistant mutants (QW1263 and QW1264) were further characterized. Both mutations did not complement each other genetically. Whole-genome sequencing-based mapping placed these mutations on the right arm of chromosome V. *In silico* complementation (Doitsidou et al., 2016) revealed that both mutants carry new alleles of *egl-9*. EGL-9 is a prolyl hydroxylase that negatively regulates the transcription factor HIF-1 (Epstein et al., 2001). These mutants possess point mutations: *egl-9(zf150)* converts His487 (CAT) to Pro (CCT), and *egl-9(zf151*) converts CAA (Gln229) to a premature TAA stop codon (**Figure 1**). The His487 residue has been previously reported as essential for the prolyl hydroxylase activity (Shao et al., 2009). Thus both mutations most likely affect the production of a fully functional EGL-9 protein.

**Figure 2:**
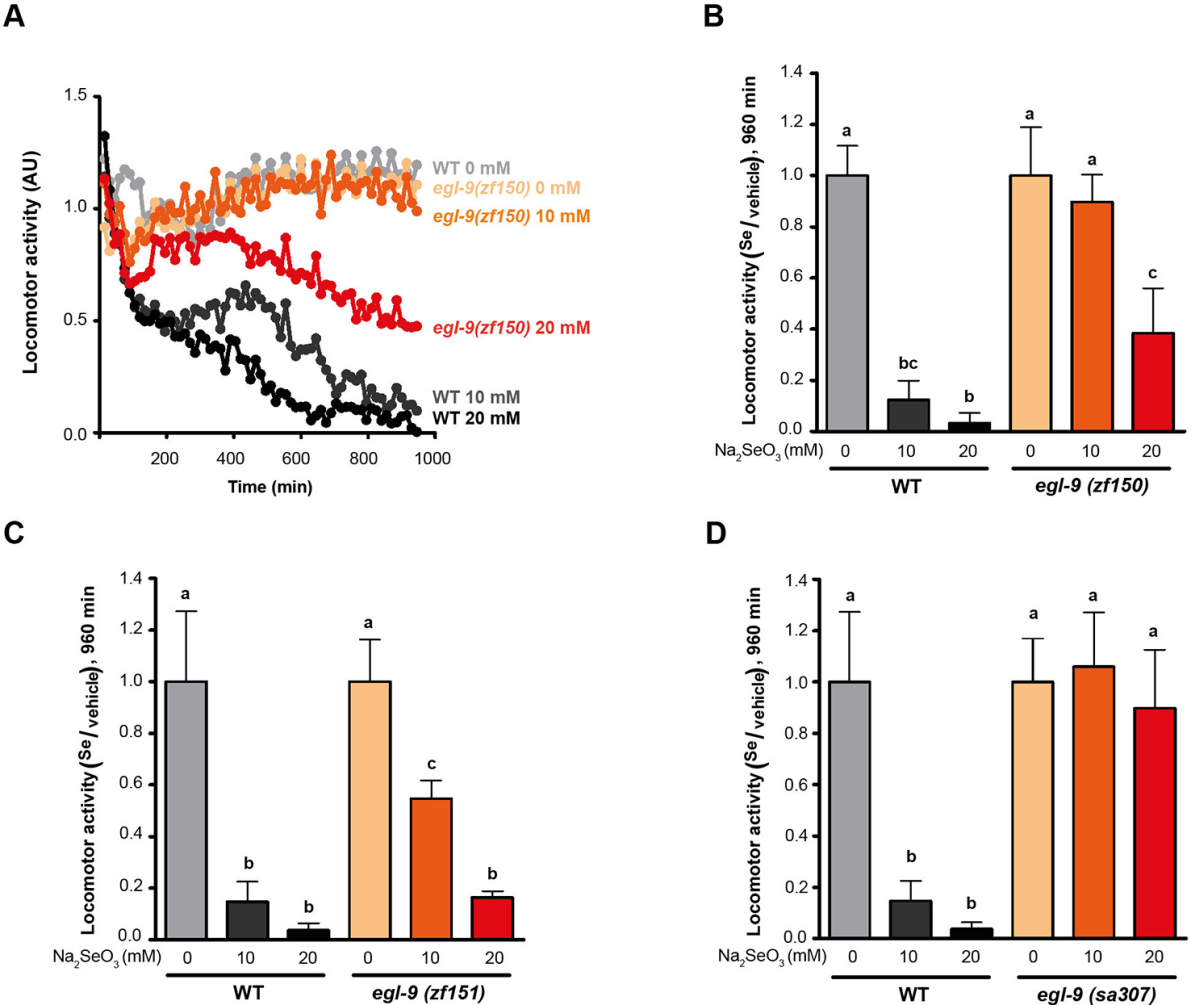
*egl-9* mutant strains are resistant to toxic selenite concentration. Locomotor activity refers to the motility of a population of worms, relative to the basal activity measured before the addition of the compound of interest, as detailed in methods. **(A)** Locomotor activity of *egl-9(zf150)* mutant worms and wild-type (WT) in 0, 10, and 20 mM of sodium selenite (Na_2_SeO_3_) for 16 h. Points indicate the average of locomotor activities measured every 15 min. AU: arbitrary units. **(B–D)** Relative locomotor activity (Se/vehicle) of *egl-9(zf150)*, *egl-9(zf151)*, *egl-9(sa307)*, and WT worms at the endpoint of incubation (16 h). Columns indicate the average locomotor activity of Na_2_SeO_3_-treated worms relative to the activity of the control without Na_2_SeO_3_ (0 mM) for each strain. Error bars (only + shown) indicate standard deviation. Variance analysis test was performed [one-way ANOVA, *p* = 1.77E-9 **(B)** and *p* = 5.86E-7 **(D)**, and Welch F test, *p* = 5.92E-5 **(C)**] followed by Tukey test. Different lowercase letters denote significant differences obtained by Tukey test (the statistical analysis and *p* values obtained are shown in **Supplementary Data**). Each graph corresponds to a representative experiment with four wells per condition per strain (80 worms per well). Three biological replicates were performed.

Since a previous report showed that *C. elegans* motility is affected with selenite in a dose-dependent manner (Morgan et al., 2010), further phenotypic analysis was carried out using an automatic motility-based assay (Simonetta and Golombek, 2007). **Figure 2A** includes typical time- and dose-dependent toxicity curves obtained using N2 and *egl-9(zf150)*. **Figures 2B–D** showed the end-point results for three strains carrying different *egl-9* alleles: QW1263 [*egl-9(zf150)*], QW1264 [*egl-9(zf151)*], and JT307 [*egl-9(sa307)*]. JT307 carries a previously reported *egl-9* loss-of-function allele (*sa307*) (Shao et al., 2009). The fact that three different *egl-9* strains were resistant to toxic Se concentrations clearly indicates that this gene is involved in Se organismal response. The mutations isolated in this study affect most, but not all, the predicted transcripts isoforms, while the JT307 strain affects all *egl-9* transcript isoforms (**Figure 1**). This could explain the difference observed in the degree of Se resistance.

### HIF-1 Controls the Organismal Selenium Response

**Figure 3:**
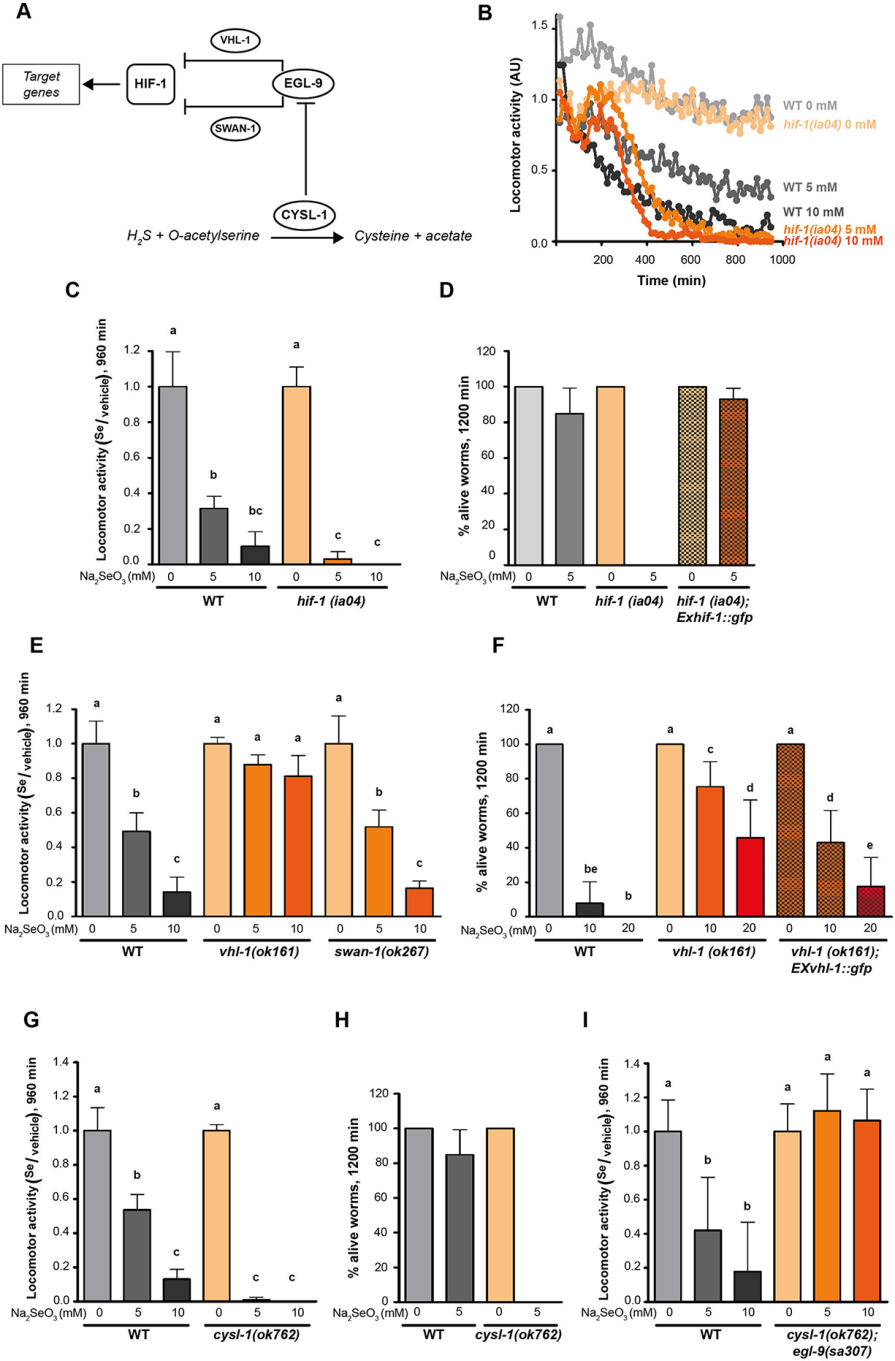
CYSL-1 functions as a selenium sensor upstream of EGL-9 leading to increased HIF-1 activity. Locomotor activity refers to the motility of a population of worms, relative to the basal activity measured before the addition of the compound of interest, as detailed in methods. Error bars (only + shown) indicate standard deviation. Different lowercase letters denote significant differences obtained by Tukey test (the statistical analysis and *p* values obtained are shown in **Supplementary Data**). **(A)** HIF-1 activation mechanism involving CYSL-1. CYSL-1 negatively regulates EGL-9 by protein-protein interaction and promotes the HIF-1 activity (Ma et al., 2012). **(B)** Locomotor activity of *hif-1(ia04)* and WT animals in 0, 5, and 10 mM of Na_2_SeO_3_ for 16 h. Points indicate the average of locomotor activities measured every 15 min. AU: arbitrary units. **(C)** Locomotor activity of *hif-1(ia04)* Na_2_SeO_3_-treated worms relative to the activity of the control without Na_2_SeO_3_ (0 mM) at the endpoint of incubation (16 h). Variance analysis test was performed (Welch F test *p* = 1.66E-5) and subsequent Tukey test. The graph corresponds to a representative experiment with four wells per condition per strain (80 worms per well). Three biological replicates were performed. **(D)** Survival of WT, *hif-1(ia04)*, and *hif-1(ia04)*; Ex*hif-1::gfp* strains in 0 and 5 mM of Na_2_SeO_3_. Columns indicate the percentage of live adult worms after 20 h of incubation. The graph corresponds to three independent experiments with one plate per strain (30–40 worms per plate). **(E)** Locomotor activity of *vhl-1(ok161)*, *swan-1(ok267)*, and WT strains in 0, 5, and 10 mM Na_2_SeO_3_ relative to the activity in 0 mM after 16 h of incubation. Variance analysis test was performed (One-way ANOVA, *p* = 2.39E-14) and subsequent Tukey test. **(F)** Survival of the WT, *vhl-1(ok161)*, and *vhl-1(ok161)*; Ex*vhl-1::gfp* strains in 0, 10, and 20 mM of Na_2_SeO_3_ after 48 h of incubation. Columns indicate the percentage of live adult worms. A Kruskal-Wallis test was performed (*p* = 2.2E-7) followed by Mann-Whitney pairwise comparisons. The graph corresponds to three independent experiments with two plates per strain (20 worms per plate). **(G, I)** Locomotor activity of *cysl-1(ok764)* **(G)** and *cysl-1(ok764); egl-9(sa307)* **(I)** mutant strains in 0, 5, and 10 mM Na_2_SeO_3_ relative to the activity in 0 mM after 16 h. Variance analysis test was performed [Welch F test, *p* = 8.53E-9 **(G)** and one-way ANOVA, *p* = 3.75E-5 **(I)**], followed by Tukey test. Each graph corresponds to a representative experiment with four wells per condition per strain (80 worms per well). Three biological replicates were performed. **(H)** Survival of WT and *cysl-1(ok762)* in 0 and 5 mM of Na_2_SeO_3_. Columns indicate the average of live adult worms after 20 h of incubation. The graph corresponds to three independent experiments with one plate per strain (30–40 worms per plate).

Since EGL-9 negatively regulates HIF-1 (Epstein et al., 2001), we examined the loss-of-function *hif-1(ia04)* mutants for its response to selenite. This strain was more sensitive than the wild-type N2 (**Figures 3B–D**). In selenite conditions *hif-1(ia04)* mutant animals significantly decreased the locomotor activity compared to the wild-type (**Figures 3B, C**). Importantly, no *hif-1(ia04)* animals survived after 20 h in selenite (5 mM), while the percentage of wild-type worms alive was greater than 80% (**Figure 3D**). These results indicated that HIF-1 is a key regulator of a Se organismal response. The expression of the *hif-1* wild-type allele in the *hif-1(ia04)* mutant strain restored the survival of worms in 5 mM selenite (**Figure 3D**), confirming the role of HIF-1 in Se response.

Two independent pathways of HIF-1 activity regulation, through VHL-1 and SWAN-1, have been described (Epstein et al., 2001; Shao et al., 2009; Shao et al., 2010). We tested strains carrying a loss-of-function alleles in these genes in response to selenite. These experiments revealed that *vhl-1(ok161)*, but not *swan-1(ok297)*, was resistant to 10 mM of sodium selenite, indicating that EGL-9 modulates HIF-1 activity through VHL-1 (**Figures 3E, F**). Most *vhl-1(ok161)* animals survived after 20 h in selenite 10 mM, while less than 10% of wild-type animals survived under these conditions. The expression of extrachromosomal *vhl-1* wild-type allele array partially rescue the wild-type phenotype (**Figure 3F**).

### CYSL-1 Is Involved in the Selenium Organismal Response

Selenium and sulfur metabolism are related. Several sulfur-metabolizing enzymes (e.g. methionine cycle and transulfuration pathway enzymes) also recognize their Se analogs (Suzuki et al., 1998; Turner et al., 1998; Bebien et al., 2001). In *C. elegans*, HIF-1 has been described to be involved in H_2_S organismal response involving the protein CYSL-1 (Budde and Roth, 2011; Ma et al., 2012). CYSL-1 catalyzes the conversion of H_2_S and acetyl serine to cysteine and acetate (Budde and Roth, 2011; Vozdek et al., 2013). However, the most relevant function described is CYSL-1 role as an EGL-9 regulator by protein-protein interaction (Ma et al., 2012) (see scheme in **Figure 3A**). In the presence of H_2_S, CYSL-1 recruits EGL-9 inhibiting its HIF-1 prolyl hydroxylase activity, operating as a sulfide sensor (Ma et al., 2012). Since hydrogen selenide (H_2_Se), a Se analog of H_2_S, is a product of selenite metabolism, we examined whether CYSL-1 is involved in Se response. A loss-of-function *cysl-1(ok762)* mutant was highly sensitive to low selenite concentrations (**Figures 3G, H**), linking CYSL-1 to Se metabolism. We then generated the double mutant *cysl-1(ok762)*; *egl-9(sa307)*, which resulted in an organism resistant to high selenite concentration (10 mM) (**Figure 3I**). This indicated that *cysl-1* acts upstream of *egl-9* and suggested a possible role for CYSL-1 as a Se sensor regulating EGL-9 activity.

*C. elegans* possesses three CYSL-1 paralogs (CYSL-2, CYSL-3, and CYSL-4), we examined mutant strains in *cysl-2* and *cysl-4*, and the RNAi of *cysl-3* worms in selenite. No differences were observed compared to N2 (data not shown).

### H_2_S Mitochondrial Oxidation Pathway Is Likely Involved in Selenium Detoxification

**Figure 4:**
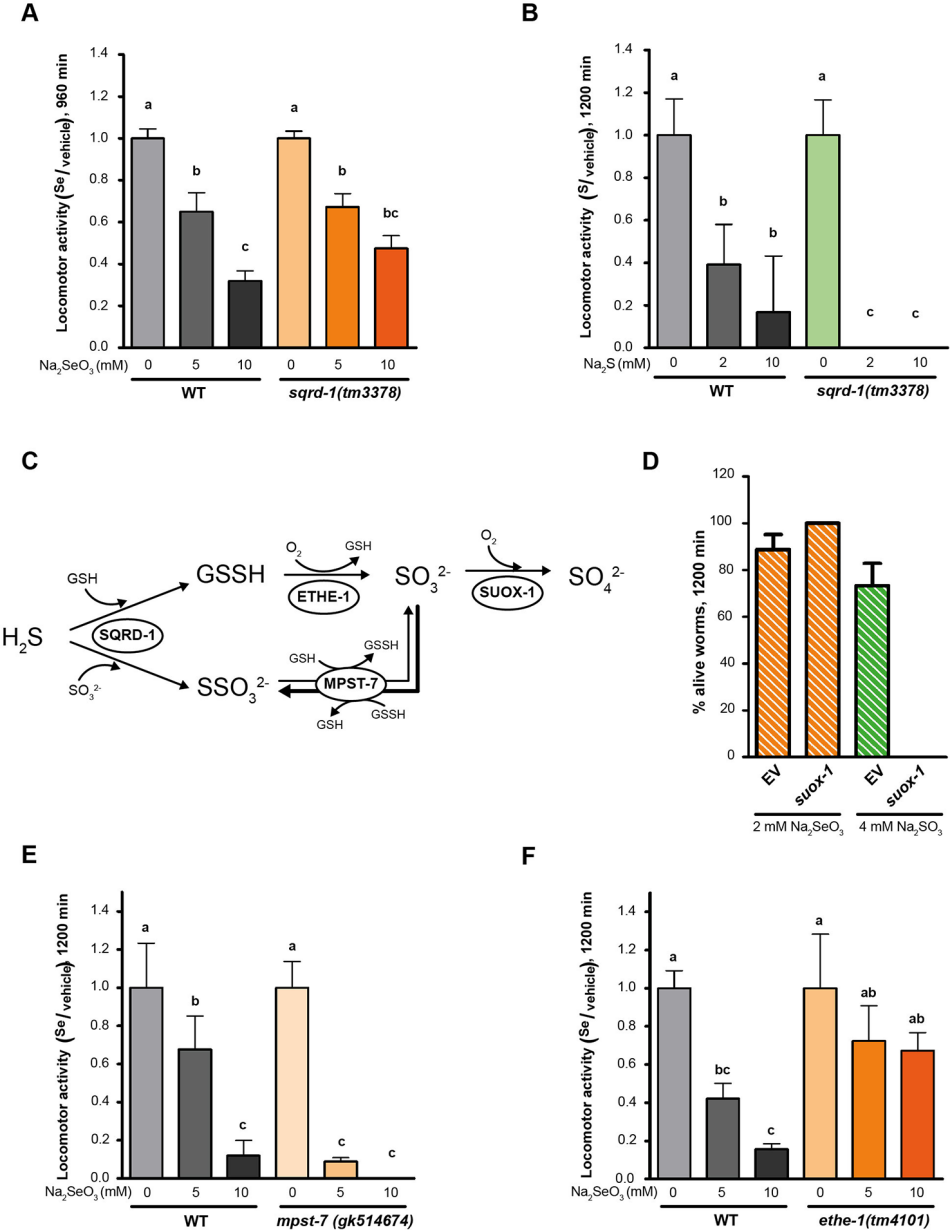
Persulfide dioxygenase (ETHE-1) and sulfurtransferase (MPST-7) are involved in selenite metabolism. Locomotor activity refers to the motility of a population of worms, relative to the basal activity measured before the addition of the compound of interest, as detailed in methods. Error bars (only + shown) indicate standard deviation, unless otherwise specified. Different lowercase letters denote significant differences obtained by *post hoc* test (the statistical analysis and *p* values obtained are shown in **Supplementary Data**). **(A)** Locomotor activity of *sqrd-1(tm3378)* and WT in 0, 5, and 10 mM Na_2_SeO_3_ relative to the activity in the control (0 mM) after 16 h of incubation. Variance analysis test was performed (Welch F test, *p* = 7.33E-14) and subsequent Tukey test. The graph corresponds to the mean of four experiments and error bars (only + shown) indicate standard error of the mean. Each experiment includes four wells per condition per strain (80 worms per well). **(B)** Locomotor activity of *sqrd-1(tm3378)* and WT in 0, 2, 10 mM Na_2_S relative to the activity in the control (0 mM) after 16 h of incubation. Variance analysis test was performed (Kruskal-Wallis, *p* = 1.08E-6) and subsequent Mann-Whitney test. The graph corresponds to one representative experiment. Each experiment includes four wells per condition per strain (80 worms per well). **(C)** H_2_S oxidation mechanism. SQRD-1 catalyzes the H_2_S oxidation. The sulfur, as sulfone, is transferred to an acceptor molecule. Glutathione (GSH) and sulfite (SO_3_^2-^) have been proposed as alternative acceptor molecules. The persulfide dioxygenase (ETHE-1) catalyzes the synthesis of sulfite using glutathione persulfide (GSSH) as precursor and the preferential reaction catalyzed by the sulfur transferase (MPST-7) is the formation of thiosulfate (SSO_3_^2−^) using sulfite as a precursor (wider line). The sulfite oxidase (SUOX-1) catalyzes the formation of sulfate (SO_4_^2−^) using sulfite (Filipovic et al., 2018). **(D)** Survival of worms of the WT strain with the *suox-1* gene expression interfered (suox-1) and the negative control (empty). Columns indicate the percentage of live adult worms after 20 h in 2 mM of Na_2_SeO_3_ and 4 mM of sodium sulfite (SO_4_^2−^). The graph corresponds to two experiments with one plate each (30–40 worms per plate). **(E**, **F)** Locomotor activity of *mpst-7(gk14674)* **(E)** and *ethe-1(tm4101)* **(F)** in 0, 5, and 10 mM Na_2_SeO_3_ relative to the activity in the control 0 mM after 16 h. Analysis of variance test was performed [Welch F test, *p* = 1.06E-5 **(E)** and *p* = 4.06E-6 **(F)**] and subsequent Tukey test. Three biological replicates were performed with similar results.

HIF-1 has been described as a master regulator of the H_2_S response in *C. elegans* (Budde and Roth, 2010). This response involves the metabolization of H_2_S by SQRD-1 (Budde and Roth, 2011). Accordingly, *sqrd-1* mutants are highly sensitive to low H_2_S concentration (50 ppm) ((Budde and Roth, 2011) and **Figure 4B**). However, exposure to selenite revealed no difference in motility or viability in *sqrd-1(tm3378)* mutant animals compared to the wild-type (**Figure 4A**). *C. elegans* possesses a SQRD-1 paralog (SQRD-2). A mutant strain in *sqrd-2* exposed to selenite did neither differ from N2 (data not shown).

SQRD-1 is the first enzyme in a sulfur metabolism pathway (**Figure 4C**), which also includes persulfide dioxygenase (*ethe-1*), a sulfurtransferase (*mpst-7*), and sulfite oxidase (*suox-1*) (Filipovic et al., 2018). We examined the role of these genes in the Se response. Since *suox-1* is an essential gene, we performed RNAi. Upon selenite exposure *suox-1* RNAi-treated animals were more sensitive to sodium sulfite than the RNAi control animals, but showed similar sensitivity to control animals in selenite conditions (**Figure 4D**). These results indicated that this enzyme is not involved in selenite detoxification. The *mpst-7(gk514674)* mutants were more sensitive to Se than wild-type animals (**Figure 4E**). In contrast, the *ethe-1* deletion mutant [*ethe-1*(*tm4101)*] was more resistant to selenite than the wild-type strain (**Figure 4F**). These results suggested that ETHE-1 and MPST-7 enzymes, and not SQRD-1 and SUOX-1, recognize Se analogs to sulfur compounds.

## Discussion

In *C. elegans* selenite exerts beneficial effects on development, cholinergic signaling, and innate immune response (Li et al., 2011; Li et al., 2014a). At high concentrations, selenite can be harmful to *C. elegans* (Morgan et al., 2010; Estevez et al., 2012). This has been proposed to result from redox imbalance and stress caused by selenite or selenite-derived species that may act as redox cyclers (Mézes and Balogh, 2009; Misra et al., 2015), and/or as a consequence of Sec misincorporation at protein Cys sites (Hoffman et al., 2019). Thus, Se species concentration must be tightly controlled.

In contrast to the well-known mechanisms of specific Sec incorporation into proteins, the organismal response to Se is not well understood. We used *C. elegans* as a model animal to assess the organismal response to this element. A screen for selenite resistant mutants identified two different strains defective in the prolyl hydroxylase EGL-9. A key target of EGL-9 is the transcription factor HIF-1, which is negatively regulated by EGL-9. Thus, we hypothesize that *egl-9* mutant animals could have constitutively high levels of HIF-1 active protein, increasing the expression of genes involved in selenite metabolism. A HIF-1 mutant was hypersensitive to selenite, supporting the role of HIF-1 in the response. HIF-1 is a key transcription factor induced by hypoxia. In addition, HIF-1 is a master gene for other stressors, driving different cytoprotective responses (Wong et al., 2013). In particular, HIF-1 is a key regulator of the adaptive response to hydrogen cyanide and H_2_S, and to pathogens such as *Pseudomonas* (Budde and Roth, 2011). EGL-9 mutants are resistant to selenite and to H_2_S, while HIF-1 are sensitive to both chemicals. *vhl-1(ok161)* mutant animals were equally resistant to selenite as *egl-9* mutants, indicating that the regulation is VHL-1-dependent, as it has been described for sulfide (Budde and Roth, 2010). Importantly, a transcriptomic survey in the presence of Se confirmed that HIF-1 target genes (e.g. *sqrd-1* and *cysl-2*) change their expression by selenite (Boehler et al., 2014).

To assess whether the HIF-1 pathway is acting in response to an oxidative stress generated by Se, we examined *hif-1(ia04)* and *egl-9(zf150)* mutant strains in the presence of two known oxidants: paraquat (methyl viologen) and menadione (Feng et al., 2001; Criddle et al., 2006). The *hif-1(ia04)* mutant strain was not more sensitive than the wild-type to oxidative stress (**Supplementary Figures 1A, C**), and the *egl-9(zf150)* mutant strain was not more resistant than the wild-type in response to these oxidants (**Supplementary Figures 1B, C**). These data, together with previous reports that the double mutant in both thioredoxin reductases is not more sensitive than the wild-type strain to selenite (Boehler et al., 2013), suggest that the EGL-9/HIF-1 response to selenite is not a consequence of an oxidative stress. In line with a Se-specific response, *egl-9(zf150)* mutant strain is resistant not only to selenite, but also to the organic Se compound selenomethionine (**Supplementary Figure 2**).

**Figure 5:**
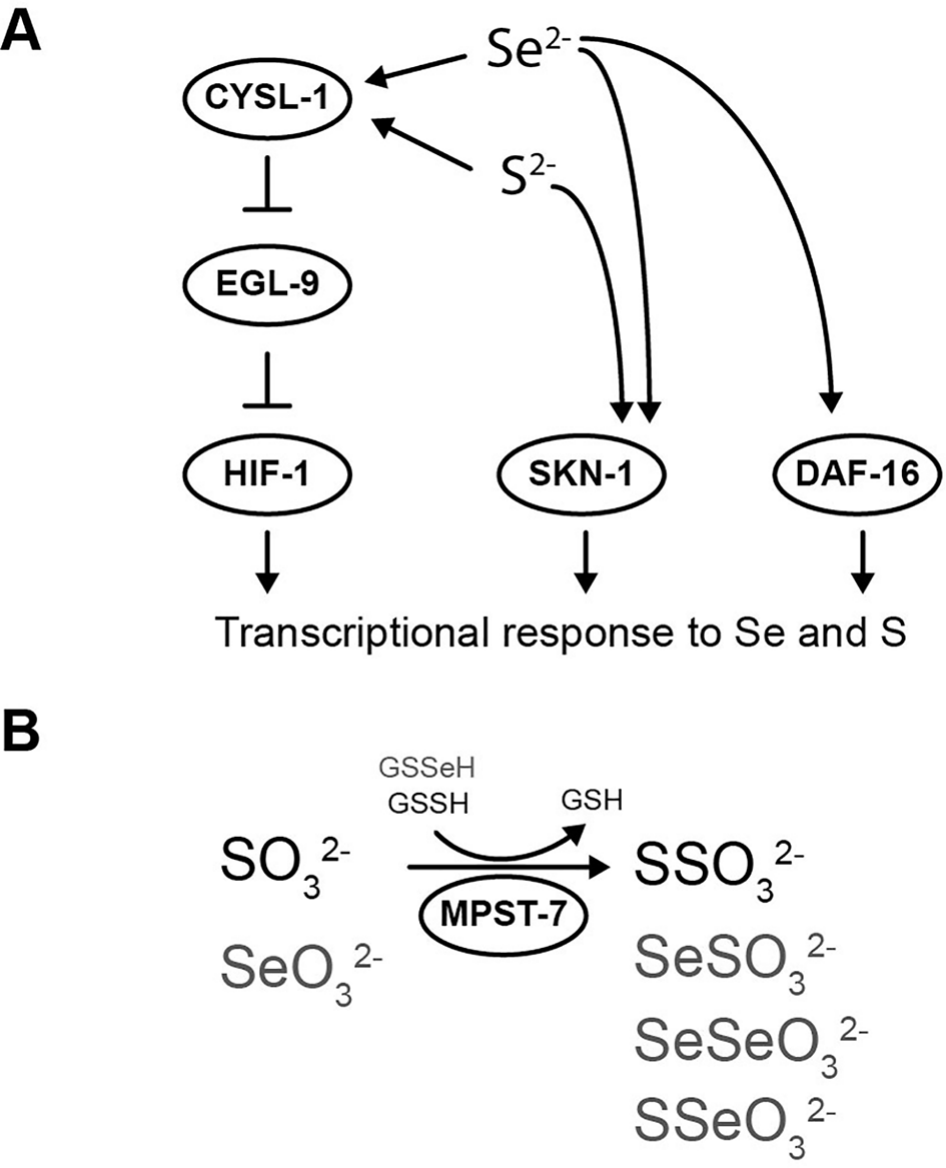
Selenium-triggered transcriptional response mechanism model and possible compounds involved in MPST-7 catalyzed reaction. **(A)** Transcriptional response to selenium involving DAF-16/*FOXO*, SKN-1/*NRF2*, and HIF-1/*HIF* has been identified. The HIF-1 pathway involves CYSL-1, which detects selenium and inhibits EGL-9 (this study). HIF-1 activation would result in a gene expression change responsible for the selenium organismal response. The same pathway was previously proposed for sulfur in reference (Budde and Roth, 2011). **(B)** MPST-7, catalyze the conversion of sulfite (SO_3_^2-^) and glutathione persulfide (GSSH) to thiosulfate (SSO_3_^2-^) and glutathione (GSH) (black) (Filipovic et al., 2018). Possible MPST-7 selenium substrates and products are shown in gray.

Similarly to HIF-1, SKN-1/*NRF2* has been described to regulate gene expression in response to selenite and sulfide (Miller et al., 2011; Li et al., 2014a). Furthermore, it has been proposed a model in which both HIF-1 and SKN-1/*NRF2* act together to coordinate a transcriptional response to sulfide (Miller et al., 2011). Additionally, the activity of SKN-1/*NRF2* in selenite conditions was also suggested in mammals. A transcriptome study in rodents with super-nutritional and toxic Se intakes revealed an expression change of multiple SKN-1/*NRF2*-target genes and a significant upregulation of EGL-9 homolog 3 (*EGLN3*) (Raines and Sunde, 2011). DAF-16/*FOXO* has also been involved in the organismal response to selenite (Li et al., 2014a; Li et al., 2014b). In selenite conditions, DAF-16/*FOXO* translocate from cytoplasm to nuclei and regulates the gene expression of DAF-16-dependent stress response genes. *daf-16(m26)* mutant strain is hypersensitive to selenite conditions and the intestinal expression of the wild-type allele ameliorate the selenite neurodegenerative effects (Estevez et al., 2014). *C. elegans* naturally lives in microbe-rich soil environments where Se levels vary. Collectively, it is possible to suggest the transcription factors SKN-1/*NRF2*, DAF-16/*FOXO*, and HIF-1/*HIF* coordinate a *C. elegans* organismal response to this element (**Figure 5A**).

Comparing with the wild-type strain, *cysl-1(ok762)* was more sensitive to selenite, while the double mutant *cysl-1(ok762); egl-9(sa307)* was more resistant. Similar to the sulfide response, the results indicated a role for CYSL-1 as a sensor of Se upstream EGL-9. However, *sqrd-1*, a HIF-1 downstream effector of *C. elegans* sulfide response (Budde and Roth, 2011), was not involved in the Se response. SUOX-1, the main sulfite detoxification enzyme (Filipovic et al., 2018), was neither relevant in the selenite response. The persulfide dioxygenase ETHE-1 and the sulfurtransferase MPST-7 showed decreased and increased sensitivity to Se, respectively. These results indicated that in contrast to SQRD-1 and SUOX-1, ETHE-1, and MPST-7 enzymes were able to recognize Se analogs to sulfur compounds. The formation of a stable Se-bound sulfur transferase in a reaction with selenite and GSH *in vitro* has been previously described (Ogasawara et al., 2001). The generation of less reactive or easily excretable Se species by this enzyme would explain the observed phenotype. A scheme showing potential reactions catalyzed by MPST-7 is shown in **Figure 5B**. Selenoglutathione persulfide (GSSeH) has been found in cell lines cultures and proposed as a Se excretion mechanism in mammals (Imai et al., 2014). The absence of ETHE-1 would lead to increased GSSeH, explaining the observed result.

In this study, we proposed a transcriptional response mediated by HIF-1 which exerts a key role in the organismal response to environmental or endogenously generated Se. The Se response pathway described has common components with the sulfide response, such as the sensor CYSL-1, EGL-9, and the transcription factor HIF-1 (**Figure 5A**). The HIF-1-target genes responsible for Se metabolization remain to be characterized. The results also suggested that sulfurtransferase and persulfide dioxygenase were involved in the Se response and indicated that effectors that deal with sulfide and Se differ.

Importantly, *egl-9* and *hif-1* are present in the human genome. Selenite has been used in diet supplements (Combs, 2001). More controversially, selenite has been used in intensive care and cancer treatments without conclusive results (Combs, 2001; Forceville, 2007; Hatfield and Gladyshev, 2009; Schomburg, 2016). The knowledge of selenite elicited pathways will contribute to understanding the organismal response to this element and its potential pharmacological use.

## Data Availability Statement

The datasets generated for this study are available on request to the corresponding authors.

## Author Contributions

LR-C performed all the experiments. MD performed the sequence analysis of the mutant strain genomes. MA provided key expertise in mutagenesis and genetic screens. MA, MD, GS and LR-C drafted the manuscript. GS and LR-C analyzed all the data, conceptualize the study and wrote the manuscript.

## Funding

CSIC Grant 2012, Universidad de la República to GS (www.csic.edu.uy). Fellowships to LR-C: POS_NAC_2012_1_8660, Agencia Nacional de Investigación e Innovación (www.anii.org.uy) and CAP_2015 CSIC Universidad de la República (www.csic.edu.uy). The funders had no role in study design, data collection and analysis, decision to publish, or preparation of the manuscript.

## Conflict of Interest

The authors declare that the research was conducted in the absence of any commercial or financial relationships that could be construed as a potential conflict of interest.
