# Supplementary material for: HIF-1 Has a Central Role in Caenorhabditis elegans Organismal Response to Selenium
Source: Front Genet. 2020 Feb 25;11:63. doi: 10.3389/fgene.2020.00063 (PMC7052493; doi:10.3389/fgene.2020.00063)
Supplement: Supplementary file 2 [file Presentation_1.pdf]

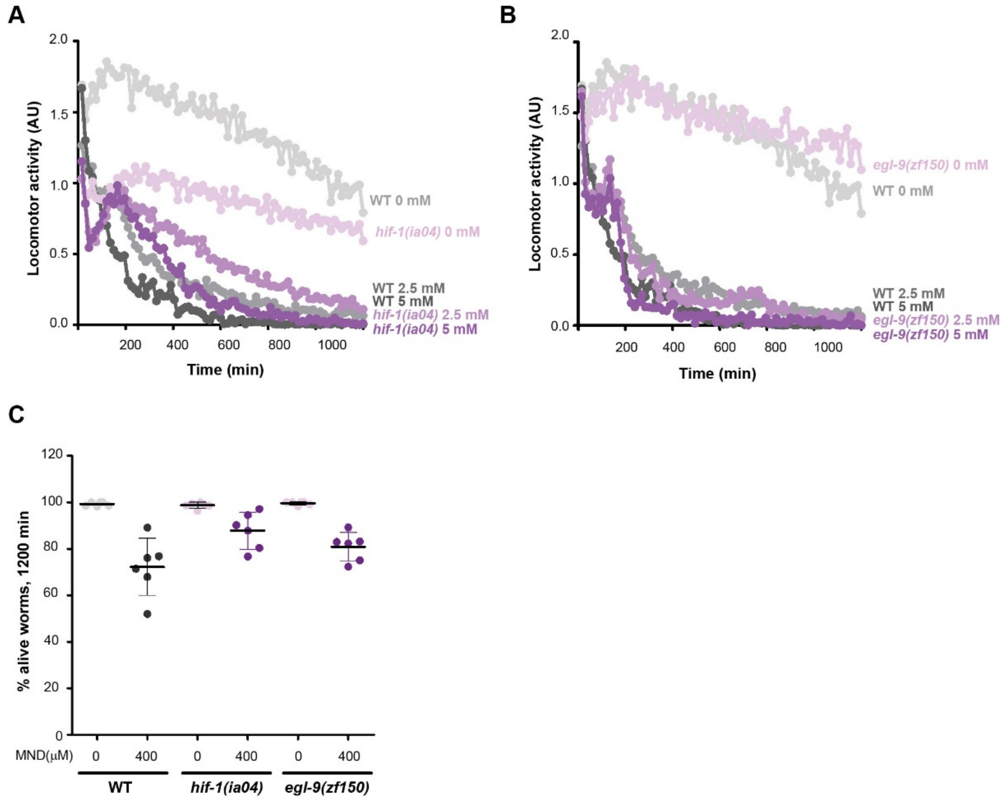

Supplemental Figure 1: Oxidative assays with paraquat and menadione. A and **B**: Locomotor activity of *hif-1(ia04)* (**A**), and *egl-9(zf150)* (**B**) in 0, 2.5 and 5 mM of paraquat (methyl viologen) for 20 h. Paraquat toxicity was assessed using the WMicrotracker^{TM} One as detailed in Methods 2.6.2. Points indicate the average of locomotor activities measured every 15 minutes. AU: arbitrary units. The graph corresponds to a representative experiment with 4 wells per condition per strain (80 worms per well). Three biological replicates were performed. The wild-type strain N2 (WT) was used as a reference. (**C**) Survival of WT, *hif-1(ia04)* and *egl-9(zf150)* strains in the 0 and 400 µM of menadione (MND). 80-100 synchronized L4 were incubated with liquid media containing the vehicle (2.3 % DMSO), 250 and 400 µM of menadione. After 20 h of incubation, 40-50 worms were transferred to NGM OP50 plates. After 4 h in NGM OP50 plates, worms alive and dead were counted. Points indicate the percentage of live adult worms per plate after 20 h of incubation. The graph corresponds to 3 independent experiments with two plates per strain (40-50 worms per plate).

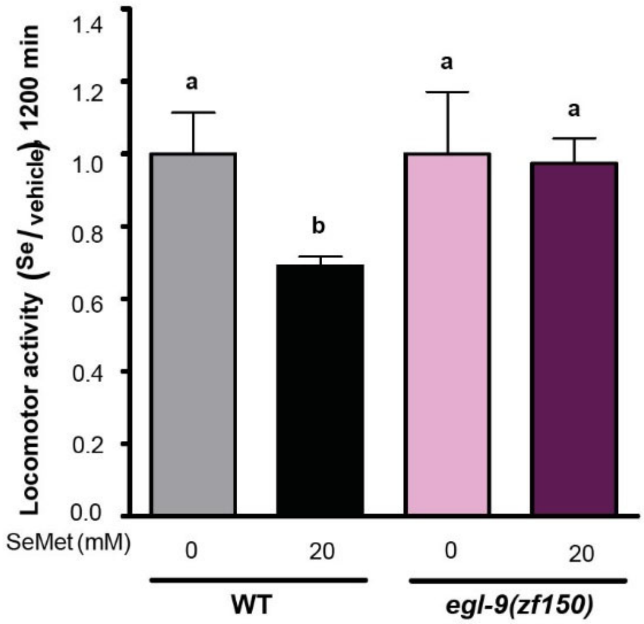

Supplemental Figure 2: Relative locomotor activity (Se/vehicle) of *egl-9(zf150)* and WT worms at the endpoint of incubation (20 h). Selenomethionine (SeMet) toxicity was studied using the WMicrotracker^{TM} One as detailed in Methods 2.6.2. Columns indicate the average locomotor activity of SeMet-treated worms (20 mM) relative to the activity of the control without SeMet for each strain. Error bars (only + shown) indicate standard deviation. Variance analysis test was performed (One-way ANOVA, p=0.004056) followed by Tukey test. Different lowercase letters denote significant differences obtained by Tukey test. The graph corresponds to a representative experiment with 4 wells per condition per strain (80 worms per well). Three biological replicates were performed.
