# Supplementary material for: HIF-1 Has a Central Role in Caenorhabditis elegans Organismal Response to Selenium
Source: Front Genet. 2020 Feb 25;11:63. doi: 10.3389/fgene.2020.00063 (PMC7052493; doi:10.3389/fgene.2020.00063)
Supplement: Supplementary file 3 [file Table_1.pdf]

Table S1. *C. elegans* strains used in this study.

Strain	Genotype	Transgene	Source
N2	*Bristol wild isolate*		*Caenorhabditis* Genetic Center
RB899	*cysl-1(ok762)X*		
RB2535	*cysl-2(ok3516)II*		
RB2436	*cysl-4(ok3359)V*		
JT307	*egl-9(sa307)V*		
ZG31	*hif-1(ia04)V*		
VC40209	*mpst-7(gk514674)V*		
CB5602	*vhl-1(ok161)X*		
RB2535	*sqrd-2(ok3516)II*		
LE436	*swan-1(ok267)V*		
TM3378	*sqrd-1(tm3378)IV*		National Bioresource Project of Japan
TM4101	*ethe-1(tm4101)IV*		
IH21	*hif-1(ia04)V; Ex[Phif-1::hif-1::gfp, Pmyo-2::mcherry]*	*ihEx1*	This study
IH23	*vhl-1(ok161)X; Ex[Pvhl-1::vhl-1::gfp, Pmyo-2::mcherry]*	*ihEx3*	
IH24	*cysl-1(ok762)X;egl-9(sa307)V*		
QW1263	*egl-9(zf150)V*		
QW1264	*egl-9(zf151)V*		
